# Ultrasound Promoted Synthesis of Bis(substituted pyrazol-4-ylcarbonyl)-Substituted Thioureas

**DOI:** 10.3390/molecules14041423

**Published:** 2009-03-31

**Authors:** Li Xiao, Chen-Jiang Liu, Yan-Ping Li

**Affiliations:** School of Chemistry and Chemical Engineering, Xinjiang University, Key Laboratory of Oil and Gas Fine Chemicals, Ministry of Education, 830046 Urumqi, P. R. China; E-mails: xiaoli56490343@163.com (L.X.), lyp_666@163.com (Y-P.L.)

**Keywords:** Thiourea, Pyrazole, Diamine, Ultrasound, Synthesis.

## Abstract

A series of novel bis(substituted pyrazol-4-ylcarbonyl)-substituted thioureas have been synthesized by the reactions of substituted pyrazol-4-ylcarbonyl isothiocyanates with different diamines under ultrasound irradiation and classical heating method at 20-25 °C. In general, substantial improvement in rates and modest yields increases were observed when reactions were carried out under sonication, compared with the classical heating method. The structures of these compounds have been elucidated by elemental and spectral (IR, ^1^H-NMR) analysis.

## Introduction

Heterocycles bearing a pyrazole moiety represent an interesting class of compounds possessing a wide spectrum of biological and pharmacological activities, such as herbicidal [1], antitumor [2], antibacterial [3], antileukemic [4], anti-inflammatory [5] properties. On the other hand, thiourea compounds have received much attention because of their wide range of biological properties such as antituberculosis [6], anticancer [7], anti-HIV [8], antimicrobial [9] activity.

**Scheme 1:**
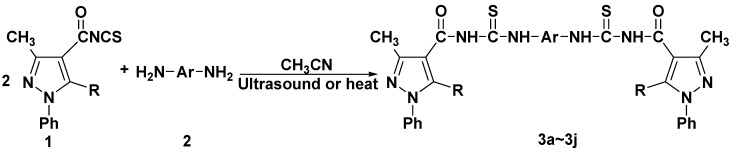
Synthesis of bis(substituted pyrazol-4-ylcarbonyl)-substituted thioureas.

The use of ultrasound in chemistry, usually known as sonochemistry, has grown spectacularly in recent years [10,11]. The success and advantages of sonochemical reactions include higher yields, shorter reaction times and milder reaction conditions when compared with traditional methods. Recently, we have reported mild and efficient procedures for the synthesis of 4-substituted pyrazolyl-3,4-dihydropyrimidin-2(1*H*)-(thi)ones under ultrasonic irradiation [12]. As a part of our interest in the synthesis of a wide range of heterocyclic systems, and in continuation of our research interest in the use of ultrasonic irradiation [12], we wish to report herein the synthesis of some novel bis(substituted pyrazol-4-ylcarbonyl)-substituted thioureas by the reaction of substituted pyrazol-4-ylcarbonyl isothiocyanates with different diamines under ultrasound irradiation (US) and classical heating conditions (CH) (Scheme 1).

## Results and Discussion

Data in Table 1 clearly show that the reactions of substituted pyrazol-4-ylcarbonyl isothiocyanates with diamines leading to bis(substituted pyrazol-4-ylcarbonyl)-substituted thioureas was carried out smoothly under both conventional and ultrasound irradiation conditions. Compared to the conventional method, the achieved yields under ultrasound irradiation increased two or three percent and the reaction times under ultrasound irradiation were dramatically shortened to 0.75 h from 7.5 h. Therefore, ultrasound irradiation exhibited some advantages over the classical condition by improving the reaction yields and reducing the reaction time. The difference in yields and reaction time (US > CH) may be a consequence of the specific effects of ultrasound.

We also examined the effect of different ultrasound irradiation frequencies on the reactions. Thus, in the case of compound **3a**, for example, when the frequency was 28 kHz, the reaction required 45 min and resulted in the formation of the desired product in 74% yield, whereas when the frequencies were 45 kHz or 100 kHz, the reaction was also complete in 45 min affording the product in 75% and 76% yield, respectively. This showed that the irradiation frequency did not significantly influence the reactions, so all the other reactions were carried out in acetonitrile under 100 kHz ultrasound irradiation.

The structures of the compounds **3a-j **were established on the basis of elemental analysis and spectral (IR, ^1^H-NMR) data. In the IR spectra of compounds **3a-j**, one sharp absorption band was seen at 1,650-1,678 cm^-1^, which belongs to the carbonyl function. The υ(NH) and υ(C=S) stretching frequencies were observed at 3,402-3,044 cm^-1^ and 1,200-1,127 cm^-1^, respectively. In the ^1^H-NMR spectra, the proton signals for compounds **3a-j** were recorded at 12.60-12.25 ppm (carbonyl band NH) and 9.84-9.40 ppm (benzene ring NH). At the same time, a singlet appearing at 2.26-2.48 ppm could be assigned to the protons of the methyl group of the pyrazole ring. 

**Table 1:** Comparison between ultrasound irradiation and conventional method.

Entry	R	Ar	Yields (%)^a^		Time (h)^b^		Mp (°C)
			A^c^	B^c^	A^c^	B^c^	
**3a**	Cl-	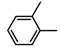	73	76	7.5	0.75	189-190
**3b**	Cl-	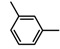	66	69	7.5	0.75	205-206
**3c**	Cl-	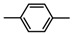	91	92	7.5	0.75	220-222
**3d**	Cl-	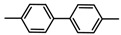	95	96	7.5	0.75	223-224
**3e**	Cl-	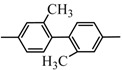	83	85	7.5	0.75	204-206
**3f**	PhO-	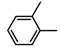	80	81	7.5	0.75	210-211
**3g**	PhO-	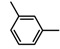	71	73	7.5	0.75	203-205
**3h**	PhO-	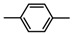	79	83	7.5	0.75	235-237
**3i**	PhO-	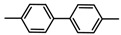	84	86	7.5	0.75	218-220
**3j**	PhO-	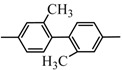	76	78	7.5	0.75	226-228

^a ^Isolated yields; ^b^ Reactions were stopped on disappearance of starting materials by TLC;

^c^ Method A: without ultrasound irradiation at 20-25 °C. Method B: under ultrasound irradiation at 20-25 °C.

## Conclusions

In summary, we have developed an efficient procedure for the synthesis of bis(substituted pyrazol-4-ylcarbonyl)-substituted thioureas under ultrasonic irradiation. We anticipate that these compounds will be subjected to biomedical screening. This work is currently in progress and the results will be reported in due course.

## Experimental

### General

Melting points were determined using a Büchi B-540 instrument and are uncorrected. The IR spectra were obtained as potassium bromide pellets with a FTS-40 spectrometer (BIO-RAD, U.S.A). The ^1^H-NMR spectra were measured in CDCl_3_ on a Varian Inova-400 spectrometer using TMS as an internal standard. Elemental (C, H, N) analysis was performed on a Perkin-Elmer Analyzer 2400. Sonication was performed in a Kunshan KQ-100VDB ultrasonic cleaner with three frequencies (28 kHz, 45 kHz, 100 kHz) and a nominal power 100 W. The reaction flask was located in the maximum energy area in the cleaner, where the surface of reactants is slightly lower than the level of the water. The reaction temperature was controlled between 20-25 °C by addition or removal of water from ultrasonic bath. Compounds **1** were synthesized according to literature methods [13].

### Synthesis of bis(substituted pyrazol-4-ylcarbonyl) substituted thioureas ***3a-3j***

*Method* *A (conventional heating):* Substituted pyrazol-4-ylcarbonyl isothiocyanates (1 mmol), diamine (0.5 mmol) and acetonitrile (25 mL) were placed in a Pyrex round bottom flask (50 mL). The mixture was stirred for a specified period at 20-25 °C. After the reaction was completed (monitored by TLC), the solids were filtered and dried at 20-25 °C. The crude mixture was recrystallized from a mixture of DMF/H_2_O. 

*Method B (ultrasonic irradiation):* Substituted pyrazol-4-ylcarbonyl isothiocyanates (1 mmol), diamine (0.5 mmol) and acetonitrile (25 mL) were placed in a Pyrex round bottom flask (50 mL). The mixture was sonicated (100 W) in a ultrasonic cleaning bath at 20-25 °C for a specified period. After the reaction was complete (as monitored by TLC), the solids were filtered and dried at 20-25 °C. The crude mixture was recrystallized from a mixture of DMF/H_2_O. Data of the compounds are shown below. 

*1,2-Di[(5-chloro-3-methyl-1-phenylpyrazol-4-yl) acylthiourea]benzene* (**3a**): white powder. IR (ν*_max._*, cm^–1^): 3,402, 3,068, 1,672, 1,161; ^1^H-NMR (*δ* ppm): 12.36 (s, 2H, NH), 9.43 (s, 2H, NH), 8.05-7.26 (m, 14H, ArH), 2.51 (s, 6H, 2xCH_3_); Anal. calcd. for C_30_H_24_Cl_2_N_8_O_2_S_2_: C, 54.30; H, 3.65; N, 16.89. Found: C, 54.21; H, 3.59; N, 16.98%.

*1,3-Di[(5-chloro-3-methyl-1-phenylpyrazol-4-yl)acylthiourea]benzene* (**3b**): flesh powder. IR (ν*_max._*, cm^–1^): 3,395, 3,044, 1,666, 1,189; ^1^H-NMR (4*δ* ppm): 12.60 (s, 2H, NH), 9.33 (s, 2H, NH), 8.26-7.26 (m, 14H, ArH), 2.61 (s, 6H, 2xCH_3_); Anal. calcd. for C_30_H_24_Cl_2_N_8_O_2_S_2_: C, 54.30; H, 3.65; N, 16.89. Found: C, 54.41; H, 3.72; N, 16.80%.

*1,4-Di[(5-chloro-3-methyl-1-phenylpyrazol-4-yl acylthiourea]benzene* (**3c**): grey powder. IR (ν*_max._*, cm^–1^): 3,401, 3,065, 1,664, 1,164; ^1^H-NMR (*δ* ppm): 12.58 (s, 2H, NH), 9.42 (s, 2H, NH), 8.11-7.25 (m, 14H, ArH), 2.54 (s, 6H, 2xCH_3_); Anal. calcd. for C_30_H_24_Cl_2_N_8_O_2_S_2_: C, 54.30; H, 3.65; N, 16.89. Found: C, 54.46; H, 3.72; N, 16.77%.

*4,4'-Di[(5-chloro-3-methyl-1-phenylpyrazol-4-yl)acylthiourea]biphenyl* (**3d**): light yellow powder. IR (ν*_max._*, cm^–1^): 3,393, 3,054, 1,672, 1,157; ^1^H-NMR (*δ* ppm): 12.62 (s, 2H, NH), 9.35 (s, 2H, NH), 7.86-7.26 (m, 18H, ArH), 2.62 (s, 6H, 2xCH_3_); Anal. calcd. for C_36_H_28_Cl_2_N_8_O_2_S_2_: C, 58.46; H, 3.82; N, 15.15. Found: C, 58.29; H ,3.76; N, 15.25%.

*2,2'-Dimethyl-4,4'-di[(5-chloro-3-methyl-1-phenylpyrazol-4-yl)acylthiourea]biphenyl* (**3e**): light yellow powder. IR (ν*_max._*, cm^–1^): 3,415, 3,084, 1,672, 1,171; ^1^H-NMR (*δ* ppm): 12.62 (s, 2H, NH), 9.35 (s, 2H, NH), 7.86-7.26 (m, 16H, ArH), 2.62 (s, 6H, 2xCH_3_), 2.42 (s, 6H, 2xCH_3_); Anal. calcd. for C_38_H_32_Cl_2_N_8_O_2_S_2_: C, 59.45; H, 4.20; N, 14.60. Found: C, 59.61; H, 4.24; N, 14.71%.

*1,2-Di[(3-methyl-5-phenoxyl-1-phenylpyrazol-4-yl)acylthiourea]benzene* (**3f**): light yellow powder. IR (ν*_max._*, cm^–1^): 3,413, 3,075, 1,678, 1,145; ^1^H-NMR (*δ* ppm): 12.49 (s, 2H, NH), 9.36 (s, 2H, NH), 7.88-7.32 (m, 24H, ArH), 2.55 (s, 6H, 2xCH_3_); Anal. calcd. for C_42_H_34_N_8_O_4_S_2_: C, 64.77; H, 4.40; N, 14.39. Found: C, 64.65; H, 4.45; N, 14.35%.

*1,3-Di[(3-methyl-5-phenoxyl-1-phenylpyrazol-4-yl)acylthiourea]benzene* (**3g**): grey powder. IR (ν*_max._*, cm^–1^): 3,392, 3,052, 1,671, 1,176; ^1^H-NMR ( *δ* ppm): 12.48 (s, 2H, NH), 9.33 (s, 2H, NH), 8.07-6.92 (m, 24H, ArH), 2.62 (s, 6H, 2xCH_3_); Anal. calcd. for C_42_H_34_N_8_O_4_S_2_: C, 64.77; H, 4.40; N, 14.39. Found: C, 64.68; H, 4.43; N, 14.29%. 

*1,4-Di[(3-methyl-5-phenoxyl-1-phenylpyrazol-4-yl)acylthiourea]benzene* (**3h**): brown powder. IR (ν*_max._*, cm^–1^): 3,412, 3,064, 1,668, 1,127; ^1^H-NMR (*δ* ppm): 12.58 (s, 2H, NH), 9.29 (s, 2H, NH), 7.89-7.56 (m, 24H, ArH), 2.58 (s, 6H, 2xCH_3_); Anal. calcd. for C_42_H_34_N_8_O_4_S_2_: C, 64.77; H, 4.40; N, 14.39. Found: C, 64.64; H, 4.36; N, 14.30%.

*4,4'-Di[(3-methyl-5-phenoxyl-1-phenylpyrazol-4-yl)acylthiourea]biphenyl* (**3i**): yellow powder. IR (ν*_max._*, cm^–1^): 3,338, 3,065, 1,650, 1,182; ^1^H-NMR (*δ* ppm): 12.53 (s, 2H, NH), 9.37 (s, 2H, NH), 7.77-6.94 (m, 28H, ArH), 2.64 (s, 6H, 2xCH_3_); Anal. calcd. for C_48_H_38_N_8_O_4_S_2_: C, 67.43; H, 4.48; N, 13.11. Found: C, 67.32; H, 4.52; N, 13.26%.

*2,2'-Dimethyl-4,4'-di[(3-methyl-5-phenoxyl-1-phenylpyrazol-4-yl)acylthiourea]biphenyl* (**3j**): yellow powder. IR (ν*_max._*, cm^–1^): 3,403, 3,072, 1,678, 1,153; ^1^H-NMR (*δ* ppm): 12.48 (s, 2H, NH), 9.45 (s, 2H, NH), 7.93-7.02 (m, 26H, ArH), 2.60 (s, 6H, 2xCH_3_), 2.43 (s, 6H, 2xCH_3_); Anal. calcd. for C_50_H_42_N_8_O_4_S_2_: C, 68.01; H, 4.79; N, 12.69. Found: C, 68.19; H, 4.85; N, 12.59%.
